# The Human Skull

**Published:** 1888-06-23

**Authors:** 


### PHYSIOLOGY AND ITS USES.

### THE HUMAN SKULL.

THE sight of a human skull with its hollow sockets and fleshless jaws makes a hysterical woman shrink; but the same sight did not prevent the gravedigger in *Hamlet* singing cheerfully at his work, and merely furnished Hamlet himself with matter for a soliloquy: "That skull had a tongue in it, and could sing once; how the knave jowls it on the ground, as if it were Cain's jawbone, that did the first murder. It might be the pate of a politician which this ass now o'er-reaches."

A physiologist no doubt acquires a certain kind of familiarity with the dead and mouldering remains of human creatures, which, if he be a man of wooden and unimaginative, intellect is apt to breed contempt. But there is no more reason why the bodily organs of the dead should become objects of irreverent disregard to him, than why the principles and dogmas of religion should afford amusement to the clergyman. The skull we hold in our hands has been part of the face of some father, or mother, or child: Let it be handled reverently; let it be spoken of soberly. It is not uncommonly called a "brain-pan," and the name is suitable enough. In adult age a human skull looks as if it might have been carved out of a single solid piece of bone. As a matter of fact it consists of no less than twenty-two distinct bones, each bearing a separate name, and each described with a painstaking minuteness by anatomists, which is the disgust and sometimes the despair of first year's students. We do not propose even to mention all these bones, much less to describe them in detail. The brain-pan proper only claims eight of them; the remaining fourteen go to make up the foundation of the most expressive of all physical structures known to man—the human face.

Although at the first glance the skull looks as if it consisted of one piece, it is quite easy even for the tyro to see and trace many of the divisions by more careful inspection. Those divisions, which are called *sutures*, have a kind of jagged look, the bones being *toothed* along their uniting margins, and fitting into each other by means of their teeth like cogged wheels. This arrangement gives a certain amount of power of expansion to the brain-pan, which is sometimes of great service to its owner, and particularly in the case of growing infants. Every reader must have seen children with enormously large heads—heads out of all proportion to the face. In these instances the bones of the head have been pushed out in all directions by a fluid exuded from the blood, vessels of the brain, producing a *hydrocephalic condition*. If there were no power of separation and expansion the pressure on the brain in such cases would prove rapidly fatal. Within certain limits nature has provided for the safety of delicate children of this class.

A very important circumstance to be remembered in connexion with the skull is that in the greater part of its extent it consists of two plates, an outer and an inner. This has a significant bearing on both practical and theoretical questions. Sometimes a man gets his head broken and is none the worse for it; at other times a broken head may set up highly dangerous symptoms or even destroy life. That is a practical aspect of the case. The so-called science of phrenology, which is a theoretical subject, is discredited among physiologists because of their knowledge of the two plates of the skull and of the cancellous bony matter between them. If the two plates were of precisely the same thickness throughout and always equally distant from each other, the question of phrenology would not be particularly touched by them. But as a matter of fact there is no scientific reliance to be placed on the bumps of the head, because they may be large or small without any reference whatever to the brain substance beneath them, but merely because the outer or inner plate of the skull may be thicker or thinner than usual at a particular point, or because the cancellous or honey-comb-like structure between them may be more or less in quantity. Phrenology therefore, as phrenologists understand it—that is, the mapping out of a man's character by means of the bumps and hollows of his head—is a perfectly impossible science. There is, however, a true brain science. Different parts of the brain have different functions, and the researches of Ferrier and other physiologists have succeeded in localising many of the functions with a good deal of precision. But the excess or defect of any intellectual or moral faculty certainly cannot be predicted from the outward conformation of the skull.

North-country farmers, when they want to tell a man he is a fool, often call him a "thickhead." They are much nearer the literal truth sometimes than they are at all aware of. Skulls vary immensely in thickness, and some men's are in parts more than half an inch thick. A blow on the head may easily crack the outer plate of the skull; and if that plate only be injured the harm may be very slight. But if the blow be such as to crack the inner plate as well, and especially if a portion of the plate be driven in upon the brain substance, very alarming and sometimes fatal consequences may follow. Modern surgery has had some triumphs in this field. Certain symptoms, which could only be produced by mechanical pressure, have been taken as indicating that the pressure was at a given point. A trephining operation—that is, the complete removal of a circular piece of bone—has enabled the pressure to be relieved and a wonderful cure to be effected. It is a bad thing to beat children about the head, even with the open hand; and they should never be struck on the head at all with hard instruments. Schoolmasters who punish boys by "boxing their ears" ought to be severely reprimanded. The internal ear is a most delicately-constructed piece of mechanism, and nature has provided for its care with the nicest minuteness. Hot-tempered teachers will do well to bear this in mind.

At the present time, when women are entering more into competition with men, a few facts as to structural differences in the sexes may help us to get at the intentions of nature with regard to them. It is a proved certainty that "the female skull is, in general, smaller, lighter, and smoother than that of the male; it is less marked by muscular prominences, and has the frontal sinuses less developed. The face is smaller in proportion to the cranium, the jaws narrower, and the frontal and occipital regions less capacious in proportion to the parietal. The female skull resembles the formed skull of the boy more than that of the adult male; but it must also be admitted that it is often impossible to determine the sex by the appearance or form of a skull."

It is a well-known and instructive fact that different races of men have different types of skull, importing very definite physical and intellectual characteristics. There are several well-defined types. Some skulls are very long from before backward; others are long from side to side; in some races the jaw is pushed forward and the forehead recedes; in others the forehead is forward and the jaw relatively drawn back. Among the smallest skulls known to science are those of the Hindoo and the ancient Peruvian; among the most massive, those of the Scandinavian, the Caffre, and the Maori. The people of savage races have several features which distinguish them from those who are more civilised, one of which is worthy of special note—viz., a greater strength of jaws and teeth, and a projection of the lower face which distinctly denotes a nearer kinship to the lower animals than that which marks the higher races. We pointed out last week that Goethe had devoted both time and critical ability to the subject of brain structure and function; and it must be admitted that the study is deeply interesting and important from the point of view of anyone who wishes to understand the world in which he lives and his own place and duty therein.

